# Birth Outcomes Associated With E-Cigarette and Non–E-Cigarette Tobacco Product Use During Pregnancy: An Examination of PATH Data Waves 1–5

**DOI:** 10.1093/ntr/ntac111

**Published:** 2022-04-26

**Authors:** Amy M Cohn, Hoda Elmasry, Robert C Wild, Amanda L Johnson, Haneen Abudayyeh, Allison Kurti, Victoria H Coleman-Cowger

**Affiliations:** Health Promotion Research Center, University of Oklahoma Health Sciences Center, Stephenson Cancer Center, Oklahoma City, OK, USA; Department of Pediatrics, University of Oklahoma Health Sciences Center, Oklahoma City, OK, USA; Independent Researcher, Ashburn, VA, USA; Health Promotion Research Center, University of Oklahoma Health Sciences Center, Stephenson Cancer Center, Oklahoma City, OK, USA; Health Promotion Research Center, University of Oklahoma Health Sciences Center, Stephenson Cancer Center, Oklahoma City, OK, USA; ERPi, Merrifield, VA, USA; University of Vermont, Burlington, VT, USA; The Emmes Company, LLC, Rockville, MD, USA

## Abstract

**Objectives:**

To examine associations of prenatal e-cigarette use to pregnancy and birth outcomes.

**Methods:**

Currently pregnant women (*n* = 1 037) from Waves 1 through 4 of the Population Assessment of Tobacco and Health Study who had pregnancy or live birth outcome data in a subsequent wave (Waves 2–5; 2013 to 2019). Weighted bivariate and multivariable models\ examined associations between past 30-day tobacco use assessed during pregnancy (any past 30-day e-cigarette use, any past 30-day non–e-cigarette tobacco use, or no past 30-day tobacco use) with adverse pregnancy (miscarriage, abortion, ectopic or tubal pregnancy, stillbirth) and birth outcomes (preterm birth, low birth weight, birth defect, placenta previa, placental abruption, pre-eclampsia) reported in the subsequent wave.

**Results:**

Approximately 1% of pregnant women reported past 30-day exclusive e-cigarette use and 3.2% used e-cigarettes and one other tobacco product. Compared to no tobacco use, past 30-day e-cigarette use (exclusive or use with another tobacco product) during pregnancy was not associated with increased odds of an adverse pregnancy or birth outcome in bivariate or multivariable models. Past 30-day non–e-cigarette tobacco use was associated with increased odds of an adverse pregnancy outcome in multivariable models, but not an adverse live birth outcome. Compared to past 30-day cigarette use, past 30-day e-cigarette use during pregnancy was not associated with lowered odds of a birth or pregnancy outcome.

**Conclusions:**

E-cigarette use during pregnancy is rare. Understanding the positive and negative impacts of pre–natal e-cigarette use on women’s health may guide public health messaging campaigns.

**Implications:**

Results showed that past 30-day e-cigarette use during pregnancy was low, with cigarette smoking remaining the most prevalent form of tobacco use during pregnancy. Current e-cigarette use during pregnancy used either exclusively or with another tobacco product, was not associated with increased risk of an adverse pregnancy, or birth outcome. A small sample size of e-cigarette users and limited information on quantity and frequency of e-cigarette use before and during pregnancy may limit conclusions. Healthcare providers may use this information when discussing the harms and consequences associated with e-cigarette and tobacco use during pregnancy.

## Introduction

Cigarette smoking during pregnancy is the leading preventable cause of poor pregnancy outcomes, but despite this has been relatively stable over the past decade,^1^ with 10–15% reporting smoking combustible cigarettes during pregnancy.^2–4^ The negative health consequences of smoking tobacco during pregnancy are wellknown.^1,5–10^ Reducing smoking in pregnancy improves the immediate and long-term health of babies by reducing the risk of preterm birth and low birthweight.^11^

Pregnant women who smoke combustible cigarettes often find it challenging to quit and may be particularly drawn to e-cigarettes due to the perception that the devices are a less risky alternative of nicotine delivery,^12^ misbelief that e-cigarettes do not contain nicotine, or because of appealing flavor options in e-liquids.^13,14^ Limited studies have assessed the acceptability and appeal of e-cigarettes among pregnant women; however, one such study found that 74% of pregnant women who have ever tried e-cigarettes perceive them to be less harmful than “traditional” cigarettes and 73% reported using e-cigarettes to help them quit smoking combustible tobacco products.^13^

Recent studies have found that e-cigarettes are the second most prevalent tobacco product used during pregnancy, following combustible cigarettes, and nearly six times as many pregnant women who are current smokers also report e-cigarette use.^15^ The Quit4 Baby trial found that nearly 18% of pregnant smokers enrolled in text-messaging smoking cessation treatment reported past 30-day e-cigarette use, and 8.4% reported past 7-day e-cigarette use.^16^ Oncken et al.found that 53% of their sample of pregnant women seeking cessation treatment had previously tried electronic cigarettes and 14% reported e-cigarette use during pregnancy, with ever users having smoked more cigarettes per day before pregnancy and also having made more previous quit attempts.^17^ A higher percentage of the sample reported previous use of e-cigarettes for smoking cessation than the use of Food and Drug Administration (FDA)-approved smoking cessation medications or nicotine replacement therapy.

The process of “smoking” e-cigarettes, often referred to as “vaping,” differs from smoking combustible cigarettes. While vaping does not involve inhalation of tar or combustion-specific harmful or potentially harmful chemicals (HPHCs), many studies have shown that the e-liquid within e-cigarettes, the aerosol created by e-cigarettes, and the aerosol delivered to the user via inhalation contains chemicals that are classed as HPHCs (eg acetylaldehyde, acrolein, formaldehyde).^18^ Some HPHCs are delivered in equal or higher levels for e-cigarettes relative to cigarettes.^19^ Furthermore, though there is no tobacco smoke, most e-cigarettes do contain nicotine, although evidence in the literature does suggest that e-cigarettes are lower risk than combustible cigarettes because they are less toxic and dependence producing.^20–22^ However, some studies have shown that nicotine can cross the placenta and affect fetal and infant development^14^; and prenatal nicotine exposure has been associated with multiple adverse consequences, including sudden infant death syndrome (SIDS).^23–25^ There remain limited data on the health impact of prenatal e-cigarette use, including dual use of e-cigarettes and combustible cigarettes, or the association of such use with negative birth outcomes.^26^

While the use of e-cigarettes among adults has risen significantly in recent years,^27–31^ there continues to be considerable uncertainty surrounding their harm reduction potential or how their use may impact the use of combustible cigarettes.^32^ Several studies have acknowledged the timely need to determine the harm potential of e-cigarettes among high-priority populations who may be highly motivated to use e-cigarettes as a method of reducing or quitting smoking,^15,17,33,34^ including pregnant women. Furthermore, there remain limited data on the health impact of prenatal e-cigarette use, including dual use of e-cigarettes and combustible cigarettes, or the association of such use with negative birth outcomes.^26^ There is an urgent need to better understand the extent to which e-cigarettes are used during pregnancy and their impact on birth and pregnancy outcomes. This paper utilized data from the Population Assessment of Tobacco and Health (PATH) Study to examine the prevalence and correlates of past 30-day exclusive e-cigarette use and dual-use of e-cigarettes with other tobacco products in a sample of currently pregnant women, and investigated associations between past 30-day e-cigarette and other tobacco use with pregnancy and birth outcomes.

## Method

Data are from the adult Waves 1 through 5 (2013 to 2019) Public Use Files (PUF) of the PATH Study, a nationally representative, longitudinal cohort study of adults in the United States, ages 18 years and older. Information on recruitment and methodology are reported elsewhere.^35^

**Figure 1.**
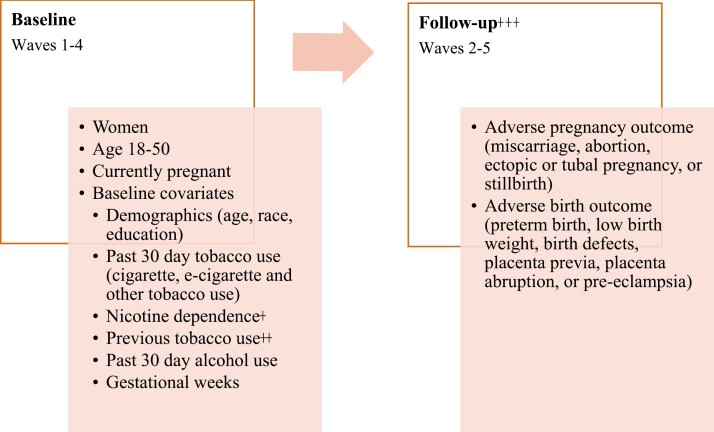
Sample for Analysis of the Association Between E-Cigarette Use During Pregnancy and Adverse Pregnancy and Birth Outcomes, PATH PUF Waves 1–5.^a^ Nicotine Dependence was Measured by the Fagerstrom Test Waking First Cigarette Score^b^ Previous Tobacco Use was Asked In Waves 3–5 Only and Captures E-Cigarette and Tobacco Use in the 3 Months Prior to the Current Pregnancy^c^ Adverse Pregnancy and Birth Outcomes Categories are Derived From the PATH Study.

The unweighted sub-sample of respondents included in the current study were 1 037 women aged 18 years or older, who were currently pregnant in Waves 1 through 4 who had a pregnancy and live birth outcome data available in the subsequent wave (Wave 2 through 5). Using an analytic approach employed in recently published PATH studies,^36,37^ each wave was considered a baseline to the subsequent wave (ie Wave 1 is a baseline to Wave 2, Wave 2 is a baseline to Wave 3, and Wave 3 is a baseline to Wave 4). Figure 1 shows a sample for analysis and variables assessed at the “baseline” wave and the “follow-up” wave.

### Measures

#### Past 30-day Tobacco Use

In the wave for which they were currently pregnant, women were asked about past 30-day use of cigarettes, e-cigarettes, traditional cigars, cigarillos, filtered cigars, hookah tobacco, pipe tobacco, smokeless tobacco, and snus. Respondents who used only e-cigarettes in the past 30days were coded as exclusive e-cigarette users; those who used e-cigarettes and at least one other tobacco product were coded as e-cigarette + other tobacco users; and those who used any tobacco product but did not use e-cigarettes in the past 30-day were coded as non-e-cigarette tobacco users. Lastly, respondents who used only cigarettes in the past 30 days were coded as exclusive cigarette users.

#### Nicotine Dependence

Respondents who were current cigarette users were asked about the time to first daily cigarette: “[On the days that you smoke, how | How] soon after you wake up do you typically smoke your first cigarette of the day? Please enter the number of minutes or hours.” Respondents were coded as using within 5 minutes (3), 6–30 minutes (2), 31–60 minutes (1), and after 60 minutes (0). Higher scores reflect stronger nicotine dependence.^38,39^

#### Tobacco Use History Prior to Pregnancy

In waves 3 and 4, currently, pregnant respondents were asked about tobacco use prior to pregnancy: “During the 3 months before your [most recent/current] pregnancy, how many cigarettes did you smoke on an average day?” In addition to cigarettes, respondents were asked about prior e-cigarettes, traditional cigars, cigarillos, filtered cigars, hookah tobacco, pipe tobacco, smokeless tobacco, and snus use (past 30-day dissolvable tobacco use was not asked in waves 3–5). Respondents who reported no tobacco use were coded as no previous tobacco use. Those who used cigarettes and/or other tobacco but not e-cigarettes were coded as “previous cigarette and/or tobacco use.” Respondents who used e-cigarettes, with or without other tobacco, were coded as “previous e-cigarette use, with or without other tobacco.” These questions were not asked in waves 1 and 2.

#### Adverse Pregnancy Outcomes

Returning women who had been pregnant during their last interview (eg, in the previous wave) were asked: “what was the outcome of your last pregnancy?,” with the response options “miscarriage, abortion, ectopic or tubal pregnancy, or stillbirth.” Using a derived variable in the dataset, women were defined as having an adverse pregnancy outcome if they endorsed at least one of the pregnancy outcomes.

#### Adverse Birth Outcomes

Returning women who had been pregnant during their last interview (eg, in the previous wave) were asked: “for your last pregnancy, did any of the following occur?,” with the response options “preterm birth (birth of baby less than 37 weeks gestational age),” “baby with low birth weight (less than 5 lbs 8 oz),” “baby with birth defects,” “ placenta previa,” “placenta abruption,” or “pre-eclampsia.” Using a derived variable in the dataset, women were defined as having an adverse birth outcome if they endorsed at least one of the birth outcomes.

#### Covariates

Covariates were assessed at the baseline wave, in which respondents were currently pregnant. Sociodemographic factors included age (18–24 years, 25–34 years, and 35+ years), race/ethnicity (non–Hispanic white, non–Hispanic black, non–hispanic other, hispanic), and education (< high school, high school diploma or General Educational Degree (GED), some college, college degree or higher). In addition, self-reported past 30-day alcohol use (yes/no) during pregnancy and gestational age (weeks) at the baseline wave were included as covariates.

For all variables, responses of “don’t know” and “refused” were treated as missing.

### Analyses

Weighted estimates examined the prevalence of past 30-day individual tobacco use among currently pregnant women at baseline (waves 1 to 4), and by adverse birth and adverse pregnancy outcomes. Next, weighted cross-tabulations examined the sample characteristics in the full sample and across three tobacco use groups: no past 30-day tobacco use, past 30-day e-cigarette use (inclusive of exclusive e-cigarette use and dual-use of e-cigarettes with at least one other tobacco product in the past 30-days), and past 30-day non-e-cigarette tobacco use. The prevalence of exclusive e-cigarette use was too low to include as a separate grouping variable. Next, generalized estimating equations (GEE) with an independent covariance, binomial distribution, and logit link function were utilized to assess the association between past 30-day use at Wave 1–4 with adverse pregnancy or birth outcomes assessed at the subsequent wave (Waves 2–5). The same three-level tobacco use indicator was used: Past 30-day e-cigarette use (exclusive use or dual use with other tobacco products in the past 30-days), past 30-day non-e-cigarette tobacco use, and no past 30-day tobacco use (reference group). Adjusted models controlled for age (at baseline), race/ethnicity, gestational age at baseline (in weeks), and past 30-day alcohol use (at baseline). Analyses were conducted using svy and xtgee commands in Stata/MP version 16. Population and replicate weights using the balanced repeated replication (BRR) method and Fay’s adjustment of 0.3 were created to adjust for the complex survey design. Analyses utilized Wave 5 all-wave weights. The percentages were weighted to represent the USpopulation.

## Results

### Prevalence of Past 30-day Tobacco Use

**Figure 2.**
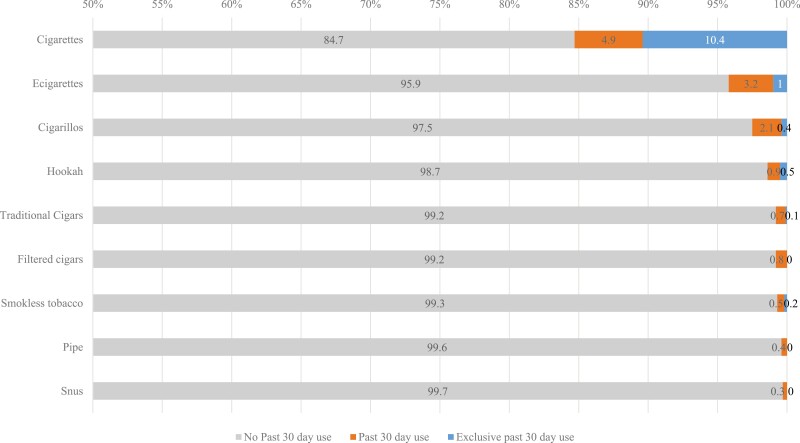
Prevalence of Past 30 days of Tobacco Use Among Currently Pregnant Women at PATH Baseline Waves 1–4, Weighted Percent.

Figure 2 shows the prevalence of past 30-day tobacco use (categories are not mutually exclusive) among currently pregnant women across each of the tobacco products assessed. Past 30-day cigarette smoking was the most prevalent form of tobacco use, reported by 15.3% of the sample, followed by past 30-day e-cigarette use (inclusive of other tobacco use), which was reported by 4.2% of currently pregnant women; 1% of whom reported exclusive use of e-cigarettes in the past 30 days. Past 30-day cigarillo use was reported by 2.5% of the sample. The remainder of the tobacco products (traditional cigars, pipe, snus) were reported by approximately 1% or less of the sample of currently pregnant women.

**Figure 3.**
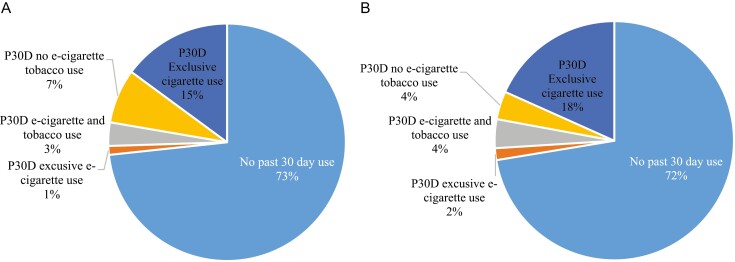
(a) Past 30 Days Tobacco Use Status Among Respondents Reporting An Adverse Pregnancy Outcome, PATH Waves 1–5. (b) Past 30-day Tobacco Use Among Respondents Reporting An Adverse Birth Outcome, PATH Waves 1–5.

Figure 3a and 3b shows that past 30-day tobacco use among women reporting adverse pregnancy or birth outcomes. Among currently pregnant women who reported an adverse pregnancy outcome, approximately 1% reported past 30-day exclusive e-cigarette use, 3.2% reported using e-cigarettes and at least one other tobacco product in the past 30-days, 7.4% reported using non–e-cigarette tobacco products in the past 30-days, and 15% reported exclusive cigarette use in the past 30 days. The distribution of past 30-days tobacco use among currently pregnant women who reported an adverse birth outcome was similar (2% exclusive e-cigarette use, 4% e-cigarette and tobacco use, 4% non–e-cigarette tobacco use, 18% exclusive cigarette use).

### Sample Characteristics

Table 1 shows the characteristics of the total sample of currently pregnant women in PATH Waves 1 through 4, as well as by tobacco use status during pregnancy. The full sample was predominantly aged 25–34years (58.3%), non–Hispanic White (58.8%), and reported some college education or higher (62.9%). Nearly 4% of the total sample reported smoking a cigarette between 31 and 60 minutes after waking. Approximately 7.6% reported alcohol use in the past 30-days during pregnancy, 5.3% reported an adverse pregnancy outcome, and 14% reported an adverse birth outcome. In waves 3 and 4, the majority of respondents reported not using any tobacco products in the 3 months prior to their current pregnancy (72.26%).

**Table 1.** Demographic, Health and Substance Use Characteristics of Currently Pregnant Women (*n* = 1 037) in PATH Waves 1–4 With Outcomes in Waves 2–5.

	No Past 30 day tobacco use ^a^*n* = 732, 82.13%	Past 30 day e-cigarette use ^b^*n* = 69, 4.14%	Past 30 day non-e-cigarette tobacco use ^c^*n* = 236, 13.73%	Total (currently pregnant respondents with outcomes in subsequent wave)*n* = 1 037
	Unweighted n, Weighted % (95% CI)	Unweighted n, Weighted % (95% CI)	Unweighted n, Weighted % (95% CI)	Unweighted n, Weighted % (95% CI)
**Demographics**				
Age				
18–24 years	319, 25.81 (22.24, 29.73)	32, 38.54 (26.34, 52.37)	116, 38.15 (31.31, 45.49)	467, 28.03 (24.72, 31.6)
25–34 years	341, 60.2 (55.37, 64.84)	29, 43.21 (29.63, 57.9)	97, 51.44 (43.72, 59.09)	467, 58.29 (54.09, 62.39)
35+ years	72, 13.99 (10.8, 17.94)	8, 18.25 (9.19, 33)	23, 10.41 (7.21, 14.81)	103, 13.68 (10.91, 17.01)
Race/ethnicity				
Non–Hispanic White	364, 56.06 (50.78, 61.21)	43, 66.51 (52.86, 77.86)	149, 72.49 (66.09, 78.09)	556, 58.75 (54.41, 62.96)
Non–Hispanic Black	118, 11 (8.44, 14.21)	8, 12.99 (5.285, 28.54)	42, 14.33 (10.22, 19.73)	168, 11.54 (9.239, 14.32)
Non–Hispanic Other	51, 7.60 (4.78, 11.89)	7, 7.87 (2.73, 20.61)	19, 3.72 (2.139, 6.384)	77, 7.08 (4.712, 10.51)
Hispanic	199, 25.33 (20.84, 30.43)	11, 12.63 (7.60, 20.27)	26, 9.46 (6.192, 14.2)	236, 22.63 (18.97, 26.77)
Education				
< High school degree	80, 7.89 (5.67, 10.88)	12, 14.38 (7.14, 26.82)	50, 17.82 (13.04, 23.87)	142, 9.52 (7.45, 12.1)
High school diploma or GED	205, 25.73 (21.33, 30.7)	24, 32.41 (21.14, 46.17)	100, 37.01 (29.53, 45.17)	329, 27.56 (23.84, 31.62)
Some college or associate’s degree	266, 31.1 (26.69, 35.87)	26, 39.35 (24.68, 56.24)	70, 30.56 (23.26, 38.98)	362, 31.36 (27.52, 35.48)
College degree +	181, 35.28 (29.69, 41.31)	7, 13.86 (5.171, 32.2)	16, 14.61 (7.649, 26.12)	204, 31.56 (26.75, 36.79)
**Substance use**				
Past 30-day alcohol				
No	684, 93.06 (89.76, 95.36)	60, 86.52 (72.09, 94.1)	215, 90.4 (84.21, 94.32)	959, 92.43 (89.67, 94.49)
Yes	48, 6.937 (4.64, 10.24)	9, 13.48 (5.90, 27.91)	21, 9.605 (5.68, 15.79)	78, 7.575 (5.509, 10.33)
Nicotine Dependence (tobacco use min after waking)				
Within 5 mins		9, 11.49 (4.66, 25.64)	44, 22.22 (15.56, 30.69)	53, 3.53 (2.372, 5.22)
6–30 mins		7, 11.23 (4.22, 26.62)	31, 12.26 (8.053, 18.22)	38, 2.15 (1.476, 3.12)
31–60 mins	1, 0.08 (.01,.55)	14, 20.09 (11.45, 32.82)	60, 22.61 (16.8, 29.72)	75, 3.999 (3.14, 5.09)
Over 60 mins		11, 17.46 (8.055, 33.8)	49, 22.57 (16.99, 29.33)	60, 3.82 (2.83, 5.14)
Tobacco use history (use in the 3 months prior to current pregnancy—Waves 3–4 only)				
No previous use	300, 86.88 (82.92, 90.04)	3, 8.07 (2.179, 25.78)	10, 12.84 (5.019, 29.1)	313, 72.26 (67.29, 76.74)
Previous cigarette and/or tobacco use	55, 9.98 (7.314, 13.47)	9, 31.27 (14.84, 54.31)	94, 81.45 (66.85, 90.53)	158, 21.87 (18.29, 25.92)
Previous e-cigarette use, with or without other tobacco	17, 2.504 (1.342, 4.627)	21, 60.64 (39.37, 78.52)	7, 5.718 (2.106, 14.6)	45, 5.361 (3.551, 8.02)
**Health characteristics**				
Baseline gestational weeks (Mean, SE)	21.63 (0.40)	17.52 (1.1)	20.96 (0.94)	21.37 (0.38)
Adverse pregnancy outcome in follow-up wave				
No	673, 95.23 (93.2, 96.68)	63, 94.26 (86.54, 97.67)	216, 91.32 (85.77, 94.84)	952, 94.66 (92.73, 96.1)
Yes	59, 4.766 (3.319, 6.8)	6, 5.739 (2.33, 13.46)	20, 8.675 (5.16, 14.23)	85, 5.343 (3.90, 7.27)
Adverse birth outcome in follow-up wave				
No	576, 82.76 (78.69, 86.19)	51, 75.57 (58.65, 87.09)	167, 68.79 (60.89, 75.74)	794, 80.54 (76.75, 83.85)
Yes	96, 12.35 (9.504, 15.9)	12, 18.69 (8.826, 35.3)	49, 22.53 (16.12, 30.56)	157, 14.01 (11.29, 17.25)

Category reflects respondents who reported not using cigarettes, e-cigarettes, or other tobacco products in the past 30 days.

Category reflects respondents who reported using e-cigarettes, exclusively or in combination with other tobacco products.

Category reflects respondents who reported using tobacco products alone or in combination, excluding e-cigarettes.

Covariates differed across past 30-day tobacco use behavior during pregnancy. Compared to the full sample, women in the past 30-day e-cigarette use group, and non–e-cig tobacco use group were characterized as being: young adults (ages 18–24 years), non-Hispanic White, and endorsing lower educational attainment. Pregnant women reporting past 30-day e-cigarette use or non-e-cigarette other tobacco use also appeared to be more nicotine dependent (eg, a higher proportion reporting tobacco use within 5 minutes after waking, or within 6–30 minutes after waking) compared to the full sample were more likely to report previous e-cigarette use, and had higher rates of past 30-day alcohol use and adverse and pregnancy outcomes. Past 30-day alcohol use was highest and gestational weeks were lowest among pregnant women who reported past 30-day e-cigarette use, compared to other groups. Furthermore, adverse pregnancy and adverse birth outcomes were highest among women who reported non-e-cigarette tobacco use, compared to the other groups.

### Loss to Follow-Up

Approximately 8% (weighted) of currently pregnant respondents were lost to follow-up (*n* = 92). There were no significant differences between those lost to follow-up and those surveyed on any of the covariates (demographics, tobacco, and substance use characteristics).

### Associations of Past 30-day Tobacco Use With Pregnancy and Birth Outcomes

Table 2 shows that past 30-day e-cigarette use was not associated with increased odds of reporting either an adverse pregnancy or adverse birth outcome in both bivariate and adjusted models, compared to no past 30-day tobacco use. Compared to no past 30-day tobacco use, past 30-day non-e-cigarette tobacco use was associated with increased odds of an adverse birth outcome in bivariate (odds ratio/OR = 2.20, 95% CI: 1.35, 3.56, *p* < .01) and multivariable models (adjusted odds ratio/AOR = 2.49, 95% CI: 1.41, 4.38, *p* < .01). Compared to no tobacco use, past 30-day non–e-cigarette tobacco use was not associated with increased risk of an adverse pregnancy outcome in bivariate and multivariable models.

**Table 2.** Crude and Adjusted Odds Ratios (OR) of the Association of Past 30-day Tobacco Use Among Currently Pregnant Women With Pregnancy and Birth Outcomes (*n* = 1 037) —PATH Waves 1–5.

	Adverse Pregnancy Outcome		Adverse Birth Outcome	
	Crude OR	Adjusted OR	Crude OR	Adjusted OR
	[95% CI]	[95% CI]	[95% CI]	[95% CI]
**Past 30-day tobacco**				
No past 30-day use	*Ref*	*Ref*	*Ref*	*Ref*
E-cigarette use	1.22 (0.43, 3.41)	0.93 (0.33, 2.56)	1.66 (0.62, 4.44)	1.88 (0.67, 5.31)
Non–e-cigarette tobacco use	1.9 (0.98, 3.68)	1.51 (0.66, 3.45)	**2.2 (1.35, 3.56)** ^**^	**2.49 (1.41, 4.38)** ^**^
**Demographics**				
Age				
18–24		*Ref*		*Ref*
25–34		0.61 (0.31, 1.22)		0.85 (0.52, 1.39)
35+		0.78 (0.31, 1.96)		0.7 (0.3, 1.62)
Race/ethnicity				
Non–Hispanic White		*Ref*		*Ref*
Non–Hispanic Black		**5.16 (2.38, 11.19)** ^***^		1.03 (0.57, 1.88)
Non–Hispanic Other		0.24 (0.03, 1.75)		0.49 (0.14, 1.8)
Hispanic		2.05 (0.8, 5.24)		0.74 (0.43, 1.28)
Education				
< High school degree		4.62 (0.99, 21.59)		0.54 (0.21, 1.4)
High school diploma or GED		2.08 (0.56, 7.64)		0.4 (0.17, 0.95)
Some college or associate’s degree		2.12 (0.56, 8)		0.96 (0.45, 2.07)
College degree +		*Ref*		*Ref*
**Substance use**				
Past 30-day alcohol				
No		*Ref*		*Ref*
Yes		1.75 (0.61, 5.02)		0.87 (0.29, 2.56)
**Health characteristics**				
Baseline gestational weeks		0.98 (0.95, 1.02)		1.01 (0.98, 1.04)

OR, odds ratio.

*p* ≤ .05.

*p* ≤ .01.

*p* ≤ .0001.

### Post-hoc Analyses

We further examined the effects of past 30-day e-cigarette use versus past 30-day cigarette use on adverse pregnancy and birth outcomes. Multivariable models were re-examined, this time including a three-level indicator capturing past 30-day tobacco use during pregnancy as no past 30-day tobacco use, past 30-day e-cigarette use (with or without other tobacco use in the past 30-days), and past 30-day cigarette smoking as the reference group (with or without other tobacco use in the past 30-days). Models controlled for age, race, ethnicity, education, alcohol use in the past 30 days, and gestational weeks. Supplementary Table 1 shows adjusted odds ratios of the association of past 30-day cigarette and e-cigarette use with pregnancy and birth outcomes. Although adjusted odds suggested that the use of e-cigarettes in the past 30-days during pregnancy was associated with a lower risk of an adverse pregnancy and adverse birth outcome compared to past 30-day cigarette use, the effects were not statistically significant. No past 30-day tobacco use was associated with significantly lower odds of reporting an adverse birth outcome relative to past 30-day cigarette use (AOR = 0.37; 95% CI: 0.02, 0.66). Women who were non–Hispanic Black (AOR = 5.4; 95% CI: 2.48, 11.76) compared to non–Hispanic White, and women who had lower education (AOR = 5.27; 95% CI: 1.02, 27.07) had significantly higher odds of reporting an adverse pregnancy outcome. No other variables emerged significant.

## Discussion

Few studies have examined the association between e-cigarette use during pregnancy on pregnancy and birth outcomes. Consistent with previous studies, estimates of current cigarette smoking were relatively high among the present sample of US pregnant women,^2,3^ highlighting that current cigarette smoking remains common during pregnancy despite overall declines in adult cigarette smoking over the past decade. In our study, approximately 15% of currently pregnant women reported past 30-day cigarette smoking and 4% used e-cigarettes in the past 30-days during their pregnancy, with at least one other tobacco product. Exclusive e-cigarette use was quite low, with 1% of the sample of currently pregnant women reporting only using e-cigarettes and no other tobacco products in the past month. In our primary analyses, past 30-day e-cigarette use during pregnancy (exclusive use or use with other tobacco products) was not associated with increased odds of reporting either an adverse pregnancy or adverse birth outcome relative to no tobacco use. In contrast, in both crude and adjusted models, past 30-day non-e-cigarette tobacco use during pregnancy was associated with a 2-fold increase in the odds of reporting an adverse birth outcome; this may have been largely driven by the relatively high rates of cigarette smoking in the sample. In post-hoc analyses, we attempted to “tease apart” the influence of pre-natal e-cigarette use versus cigarette use on the outcomes of interest. Results indicated that other, past 30-day e-cigarette use and cigarette smoking were not associated with increased risk of reporting either an adverse birth and pregnancy outcome, after controlling for demographics. The latter finding was surprising, as most literature shows that cigarette smoking during pregnancy is linked to poor pre- and postnatal health outcomes.^6,8–10^ Because most e-cigarette users in the sample also reported using another tobacco product, this may also partially explain why there were no differential effects of e-cigarette compared to cigarette use on the outcomes of interest in post hoc analyses. It is also possible that women using e-cigarettes were not using other tobacco products as frequently or as heavily before pregnancy, and may not have used e-cigarettes as frequently or heavily compared to non–e-cigarette tobacco users during pregnancy. Unfortunately, due to the nature of the PATH public-use data files, questions about the quantity and frequency of e-cigarette use were not available.^40^ We also note that relatively small number of respondents reporting any past 30-day e-cigarette use during pregnancy (*n* = 69) may have also contributed to the lack of significant findings between e-cigarette use during pregnancy and birth and pregnancy outcomes. Lastly, our decision to assess past 30-day use behavior provided only a “one time” snapshot of behavior and not an assessment of consistency or quantity of smoking throughout pregnancy.

E-cigarettes were the second most prevalent tobacco product used in the past 30 days among currently pregnant women. Based on findings from the current study, the use of e-cigarettes exclusively or with another tobacco product in the past 30-days during pregnancy was unrelated to increased the risk of adverse pregnancy or birth outcomes in comparison to cigarette smoking. We note that a lack of statistical significance may not necessarily be indicative of lower risk and could be due to a variety of reasons, including small sample size, limited information about heaviness and frequency of e-cigarette and other tobacco use both before and during pregnancy, and our measurement of e-cigarette use that included both exclusive users and dual users of other tobacco products. Regardless, this is the first study of which we are aware that has used US national data to examine associations of e-cigarette use and other tobacco use, during pregnancy, with birth and pregnancy outcomes. This information could help clinicians educate pregnant women about the potential risks associated with prenatal e-cigarette use in several ways. First, pregnant women’s use of e-cigarettes may indicate an interest in quitting smoking. As such, screening for e-cigarette use during pregnancy could provide a foundation for providers and patients to talk about evidence-based cessation methods and other resources to facilitate quitting.^41,42^ Second, it is possible that pregnant women who use e-cigarettes are unaware of the effects of nicotine on the developing fetus. Providers who treat pregnant patients should be clear to reiterate the impacts of nicotine ingestion on the development of an unborn baby, and postnatal developmental outcomes. Unfortunately, the PATH survey does not explicitly ask respondents to rate their knowledge of potential harms to fetuses from using e-cigarettes during pregnancy. Future iterations of US surveillance studies should consider including questions that ask about knowledge and awareness of e-cigarette harms during pregnancy, on both the baby and the mother, and whether different nicotine concentrations in e-cigarettes used during pregnancy may have an impact on outcomes.

Several limitations should be noted. First, even though this study used data from a US nationally representative sample, because the sample size of currently pregnant women was small, we were unable to examine exclusive e-cigarette use as a unique correlate of adverse pregnancy and birth outcomes, dissociate individual adverse pregnancy and birth outcomes from each other or examine unique contributions of illicit drug use and nicotine dependence during pregnancy on these outcomes. Second, we were unable to assess the frequency and quantity of e-cigarette or other tobacco use (eg, heaviness of use) during pregnancy, history of use earlier in pregnancy or prior to pregnancy in all waves of data, or biomarkers of exposure from tobacco product use. Biomarkers of exposure across tobacco use groups might differ and predict the outcomes of the interest. Furthermore, underreporting of tobacco use during pregnancy may be possible, highlighting the need to examine biomarkers of tobacco use exposure as predictors of pregnancy and birth outcomes rather than relying solely on self-report. Additionally, we acknowledge that any past 30-day use might capture very light e-cigarette or other tobacco use, and therefore underestimate the associated risks. Because we were unable to include an assessment of the patterns of e-cigarette use before and during pregnancy, it is unclear whether e-cigarette use in our sample represents the lower or higher end of e-cigarette-related risk. Information on the consistency in tobacco use across pregnancy was not captured, as PATH only queried respondents once per year. It may be that cigarettes smokers are smoking multiple cigarettes per day and have been for years, while e-cigarette users may only use on occasion and may have started during pregnancy or early on in their pregnancy. Lastly, the common criterion for assessing tobacco use behavior during pregnancy across studies may help generalize study findings. For example, our study showed the prevalence of current cigarette use among pregnant women was 15%, a number slightly higher than other US studies.^2,3,15^ This difference may be attributable to how current use was defined in our analysis. A previously published study using Wave 1 of the PATH study showed that 12.8% of currently pregnant women reported current cigarette smoking,^15^ but this study defined current use as ever use of cigarettes “fairly regularly” and currently using “some days or every day use,” or having used the product in the past but “not fairly regularly.” It is possible that our definition of past 30-day use increases the estimate of use.

## Conclusions

Results from our study show that e-cigarette use during pregnancy remains low and the current smoking rate among pregnant women in the United States is still high. This suggests that current approaches to smoking cessation targeting pregnant women could be improved. Prenatal care is an opportune time to provide evidence-based care for smoking cessation intervention. E-cigarette use during pregnancy may be indicative of a desire to reduce or quit conventional cigarette smoking, and given the greater likelihood of dual use with other tobacco products and the known negative health consequences of nicotine use on fetal development, it is important for clinicians to assess and explore e-cigarette use during pregnancy and provide education utilizing a similar approach to that currently used for combustible cigarette smoking. Given the study’s limitations, we cannot conclude that e-cigarette use during pregnancy confers greater or lower risk to the mother or unborn fetus relative to cigarette smoking or other tobacco use, or that e-cigarettes are a less risky alternative to cigarette smoking during pregnancy. E-cigarettes are a relatively new technology and they are rapidly evolving, as are their patterns of use. Future research should dissociate the impacts of exclusive e-cigarette use from dual use on pregnancy and birth outcomes among larger samples of pregnant women, examine differences in biomarkers of exposure between e-cigarette and non–e-cigarette tobacco user groups of pregnant women, and the potential impact of biomarkers of exposure on pregnancy and birth outcomes.

## Supplementary Material

A Contributorship Form detailing each author’s specific involvement with this content, as well as any supplementary data, are available online at https://academic.oup.com/ntr.

ntac111_suppl_Supplementary_Table_S1

ntac111_suppl_Supplementary_Taxonomy-form

## Data Availability

All data are publicly available.
