# Supplementary material for: Birth Outcomes Associated With E-Cigarette and Non–E-Cigarette Tobacco Product Use During Pregnancy: An Examination of PATH Data Waves 1–5
Source: Nicotine Tob Res. 2022 Apr 26;25(3):444–52. doi: 10.1093/ntr/ntac111 (PMC9910157; doi:10.1093/ntr/ntac111)
Supplement: ntac111_suppl_Supplementary_Table_S1 [file ntac111_suppl_supplementary_table_s1.docx]

Supplemental Table 1. Adjusted odds ratios of the association of past 30-day cigarette and e-cigarette use among currently pregnant women with pregnancy and birth outcomes (n = 1,009), PATH Waves 1-5.

|  |  | **Adverse Pregnancy Outcome** | **Adverse Birth Outcome** |
| --- | --- | --- | --- |
|  |  | **Adjusted OR (95% CI)** | **Adjusted OR (95% CI)** |
| **Past 30-day tobacco use^+^** | |  |  |
|  | No past 30-day use | 0.60 (0.26, 1.42) | **0.37 (0.02, 0.66)**** |
|  | E-cigarette use, with or without other tobacco | 0.53 (0.16, 1.77) | 0.69 (0.25, 1.89) |
|  | Cigarette use, with or without other tobacco use | *Ref* | *Ref* |
| **Demographics** | |  |  |
| Age |  |  |  |
|  | 18-24 | *Ref* | *Ref* |
|  | 25-34 | 0.58 (0.29, 1.17) | 0.83 (0.51, 1.36) |
|  | 35+ | 0.75 (0.3, 1.9) | 0.67 (0.29, 1.56) |
| Race/ethnicity | |  |  |
|  | Non-Hispanic White | *Ref* | *Ref* |
|  | Non-Hispanic Black | **5.4 (2.48, 11.76)***** | 1.12 (0.6, 2.09) |
|  | Non-Hispanic Other | 0.26 (0.04, 1.9) | 0.51 (0.14, 1.9) |
|  | Hispanic | 1.96 (0.75, 5.11) | 0.77 (0.45, 1.34) |
| Education | |  |  |
|  | < High school degree | **5.27 (1.02, 27.07)*** | 0.53 (0.2, 1.36) |
|  | High school diploma or GED | 2.48 (0.6, 10.32) | 0.4 (0.17, 0.97) |
|  | Some college or associate's degree | 2.5 (0.59, 10.5) | 0.96 (0.44, 2.09) |
|  | College degree + | *Ref* | *Ref* |
| **Substance use** | |  |  |
| Past 30-day alcohol | |  |  |
|  | No | *Ref* | *Ref* |
|  | Yes | 1.96 (0.68, 5.66) | 0.87 (0.29, 2.67) |
| **Health characteristics** | |  |  |
| Baseline gestational weeks | | 0.98 (0.95, 1.02) | 1.01 (0.99, 1.04) |

+ Excludes exclusive other tobacco use

*p≤0.05, **p≤0.01, ***p≤0.0001
